# Supplementary material for: Active site specificity profiling datasets of matrix metalloproteinases (MMPs) 1, 2, 3, 7, 8, 9, 12, 13 and 14
Source: Data Brief. 2016 Feb 22;7:299–310. doi: 10.1016/j.dib.2016.02.036 (PMC4777984; doi:10.1016/j.dib.2016.02.036)
Supplement: Supplementary file 10 — Supplementary material [file mmc10.zip › WebPICS_hMMP9_G_1%/P3.html]

 

PICS results


|  |  |
| --- | --- |
| **P3\_A**  22 in 146 sites   15.1 %    effects > 10 perc. pnts.  (vice-versa in brackets)  P1\_A: 15.4 (14.1)   P1prime\_I: 12.2 (12.2)   P2prime\_L: 10.4 (12.7)   P3prime\_G: 28.8 (20.4)   P3prime\_S: -16.7 (-11.9) |  |
| **P3\_V**  13 in 146 sites   8.9 %    effects > 10 perc. pnts.  (vice-versa in brackets)  P1prime\_Q: 14.9 (16.1)   P2\_A: 25.5 (17.4) |  |
